# Intuition in chess: a study with world-class players

**DOI:** 10.1007/s00426-023-01823-x

**Published:** 2023-04-18

**Authors:** Philippe Chassy, Rick Lahaye, André Didierjean, Fernand Gobet

**Affiliations:** 1 Department of Psychology, University of Liverpool, Liverpool, UK; 2 ChessZebra, Rotterdam, The Netherlands; 3 Université de Bourgogne-Franche-Comté, Besançon, France; 4 London School of Economics and Political Science, London, UK

## Abstract

Intuition plays a central role in cognition in general and expertise in particular. Dreyfus and Dreyfus’s (1986) and Gobet and Chassy’s (2008) theories of expert intuition propose that a characteristic feature of expert intuition is the holistic understanding displayed by experts. The ideal way to test this prediction is to use highly expert participants and short presentation times. Chess players (*N* = 63), ranging from candidate masters to world-class players, had to evaluate chess problems. Evaluating the problems required an understanding of the position as a whole. Results demonstrated an effect of skill (better players had better evaluations), complexity (simpler positions were better evaluated than complex positions) and balance (accuracy diminished when the true evaluations became more extreme). A regression analysis showed that skill accounted for 44% of the variance in evaluation error. These important results support the central role of holistic intuition in expertise.

## Introduction

The role of intuition and analytical thinking is a central question for our understanding of cognition in general and expertise in particular. When facing a problem, such as finding a patient’s correct diagnosis in medicine, do novice and expert humans produce a solution rapidly by intuition, or do they need to use slower thinking methods such as deliberation and reasoning? In his seminal study of chess players, De Groot (1978, 1986; De Groot et al., 1996) argued that intuition is omnipresent in thinking, although it is often supplemented by conscious heuristics and analysis of the problem at hand. In contrast, Kahneman (2011) proposed a dual-system theory, where system 1 is rapid, unconscious and intuitive and system 2 is slow, conscious and analytical. System 1 operates initially; if it fails, system 2 enters into action. (For a discussion of alternative orders for these two systems, see for example de Neys & Pennycook, 2019.)

Gobet and Chassy (2008, 2009) proposed five key characteristics of expert intuition: rapid perception of the key features of a situation, lack of awareness of the way decisions are reached, the presence of emotions, holistic grasp of the situation, and the presence of decisions that are correct more often than chance. Whilst most authors agree that expert intuition is a genuine phenomenon, others (e.g., English, 1993; Holding, 1985; Montero & Evans, 2011) have argued that the role of intuition has been overrated at the expense of more conscious thinking. Whatever their view, authors concur that intuition is a phenomenon that has been difficult to document unambiguously with empirical evidence and that the best way to study expert intuition is to use tasks with short presentation times and highly skilled participants.

In a series of seminal publications, Simon (Chase & Simon, 1973; Newell & Simon, 1972; Simon, 1995) argued that expertise is a combination of pattern recognition and selective search through the states of a problem space. For him, intuition can be equaled to pattern recognition. A similar theory was proposed by Klein (2003) in his recognition-primed decision model, which also emphasizes pattern recognition and selective search. In contrast, Dreyfus and Dreyfus’s (1986) influential five-stage theory of expertise proposed that expertise *is* intuition: experts rarely deliberate with ordinary tasks (for a discussion, see Dreyfus & Rousse, 2018; Gobet, 2018). An important difference between Simon’s theory and Dreyfus and Dreyfus’s theory is that the former assumes that the problem situation is encoded as relatively small perceptual patterns, known as chunks, whilst the latter emphasizes that experts have a holistic understanding of the problem at hand. To reconcile this difference, Gobet and Chassy (2009) proposed a theory of intuition based on template theory (Gobet & Simon, 1996). The central new assumption is that, with experts, some frequent chunks develop into templates, which are schema-like structures where information can be encoded rapidly, in about 250 ms. Recognition of patterns of chess pieces on the board makes it possible to access potentially useful information, such as the kind of moves to play, strategic ideas and tactical motifs. The extended theory also provides mechanisms explaining how emotional responses get associated with chunks and templates during practice and study, and how they might be activated in later games. Pattern recognition and emotions are two characteristic features of intuition.

Whilst the template theory of intuition maintains many aspects of Simon’s earlier theory, including the emphasis on pattern recognition and selective search, the presence of large templates also makes it possible to account for the Gestalt-like quality of intuition. Gobet and Chassy (2009) present computer simulations with CHREST, the computational embodiment of template theory, that show how the model incrementally constructs a representation of the position in memory, while reaching a holistic representation by the end of the presentation time. A possible biological implementation of the theory was proposed by Chassy and Gobet (2011).

## Studying intuition with chess

Much research on expertise has been carried out on chess, because of several favorable features offered by this domain, including strong external validity; strong ecological validity; and presence of a precise scale which quantifies players’ level of expertise (Gobet, 1993, 1998b). A first group of studies on chess intuition have focused on choice of actions (moves in chess) using protocol analysis. De Groot (1978) found that players of all skill levels displayed a highly selective search and that the best players rapidly direct their attention to good moves. Moxley et al. (2012) were interested in how players can improve on their initial choice with additional search. Players were allowed to think about the position for 5 min. Consistent with De Groot’s data, there were already skill differences with the first move proposed but, with complex positions, all players tended to improve their choice of move with additional thinking time. A limitation of these two studies is that the moves were proposed after what De Groot (1978) called the *first phase*, where players orient themselves in the position. Although its duration is affected by the type of position used, this phase typically lasts a couple of minutes. For example, it lasted 3.1 min on average in de Groot’s (1978) sample and 2.3 min in Gobet’s (1998a) sample. Another limitation of Moxley et al.’s (2012) study is that relatively little of the variance in skill was accounted for: 7.8% by the quality of the first move mentioned and 15.2% by the quality of the move ultimately chosen. This is much less than in previous studies (e.g., the quality of the chosen move accounted for 29.2% of the variance in skill in Gobet, 1998a).

More relevant for measuring initial intuitions are experiments where players must make decisions rapidly. Calderwood et al. (1988) found that masters but not class B players maintained the quality of their moves in speed chess (about 5 s per move) as compared with standard chess (about 120 s per move). In a similar vein, Campitelli and Gobet (2004) found a large skill difference in a find-the-best-move task where positions were presented for 5 s. With simpler positions, Jastrzembski et al. (2006) and Chassy and Gobet (2013) showed that threats such as the presence of a check were rapidly identified by skilled players.

It could be argued that the choice of move, albeit an eminently ecological measure, is a measure of analytical thinking that does not assess the global understanding of a position, which is a more holistic process. Some studies have addressed this issue. Holding (1979), who asked players to evaluate chess positions, found that better players proposed more correct evaluations than weaker players. Charness (1981), who required players to evaluate endgame positions, found that better players proposed better evaluations. While suggestive, these two studies suffer from two important limitations. First, the times were fairly long for studying intuition (3 min with Holding, 1979, and 10 s with Charness, 1981). Second, the skill level was low in both cases, the best players being Class A players. Thus, these two studies did not include expert players (Elo ≥ 2000).

## Complexity in chess

Chess and other board games have offered a fertile ground for studying complexity, in particular in computer science and artificial intelligence. Based on the size of the problem space, different measures of computational complexity have been developed, which depend on the average branching factor (number of possible moves) and average length of a game (Allis, 1994; Gobet et al., 2004). De Groot et al. (1996) used Shannon’s (1948) information theory to estimate the amount of information in a position likely to occur in a master game and obtained an upper limit of 50 bits; this is much lower that the amount of information in a legal, but possibly random chess position, estimated as 143 bits.

All these measures take into account the average branching factor, which is a function of the number of pieces in a position. Therefore, in the experiment below, we defined complexity using the number pieces. While it is possible to find positions with many pieces that are simple, on average this definition is valid and is reasonable with the set of positions we used. Our definition is in line with Chassy and Gobet’s approach (2013), which defines complexity as the combination of units and their interactions in a position. These properties provide a measure of the number of potential future states that the systems can take. In chess, the number of pieces constitutes an excellent indicator of complexity as it reflects both static complexity, that is the number of visual items that the perceiver has to recognize and integrate in their representation of the problem situation, and dynamic complexity, that is the number of potential moves that could be played. The dynamical evolution of a chess game cannot display aleatory properties, as random moves would be immediately punished, but it can be cyclic (draw by repetition) and includes high levels of uncertainty linked to the non-cooperative behavior of the opponent. It is because the size of the problem space is well beyond human cognitive capabilities that players rely on intuition.

## Aim of the present study

Intuition is the response of the cognitive system to a situation that overwhelms its capacity for exhaustive analysis. The five-stage theory of expertise (Dreyfus & Dreyfus, 1986) and template theory (Gobet & Chassy, 2008, 2009) propose that holistic understanding characterizes experts’ intuition. Choice-of-move tasks are unsuitable to test this prediction, since some situations can be solved by looking at only part of the problem (e.g., see Fig. 14.9 of Reingold & Charness, 2005). Evaluation tasks are better suited, as they provide an overall measure of the understanding of the whole problem (Euwe, 1998). Note that chess players are very familiar with evaluation tasks, which they carry out often when playing a game and during practice. Entire books, such as that written by world champion Max Euwe (1998), are devoted to help players evaluate positions better. In our study, intuition is operationalised by asking players to evaluate positions with no obvious solutions (i.e., checkmate) within only 5 s. Given the short time available, players cannot carry an extensive analysis of potential variations and so they must rely on their initial evaluation of the position.

The first aim of the present study, then, was to test the prediction of these two theories that strong players will evaluate chess positions better than weak players despite short presentation time. The second aim was to quantify the relation between skill and intuition by estimating the amount of variance in evaluation quality accounted by skill. While both theories emphasize experts’ holistic understanding of a problem, an important difference between the two theories concerns the way such an understanding is reached. Dreyfus and Dreyfus (1986) stress that at no point do experts identify subcomponents, while Gobet and Chassy (2008, 2009) stress that local mechanisms using fairly small components (chunks) incrementally lead to a holistic representation of a problem. Thus, a divergent prediction between the two theories is that the former predicts no complexity effect, while the second does, as it takes more time to identify chunks and templates with complex positions. The third aim was thus to test these diverging predictions. The fourth and final aim was to test whether balance (i.e*.*, which side has the advantage) affected position evaluation. This aim was exploratory in nature.

## Method

### Participants

Sixty-three players (15 females) with normal or corrected-to-normal vision completed the computer-based experiment. The mean age was 30.52 years (SD = 9.91 years). Based upon their Elo rating,^1^ the participants were assigned to one of four levels of expertise: candidate masters (1999 < Elo < 2200), masters (2199 < Elo < 2400), international players (2399 < Elo < 2600) and World-class players (Elo > 2599) players. Eight players had more than 2700 Elo, including one player with a rating above 2800 Elo. At the time of data collection, only 39 players worldwide had 2700 Elo or more. The four groups had significantly different levels of skill, *F*(3,59) = 250.86, *p* < 0.001: candidate masters (*M* = 2106.43 Elo, SD = 66.67 Elo, *n* = 14), masters (*M* = 2314.13 Elo, SD = 56.27 Elo, *n* = 15), international players (*M* = 2485.71 Elo, SD = 64.78 Elo, *n* = 17), and World-class players (*M* = 2690.94 Elo, SD = 58.82 Elo, *n* = 17). The level of expertise was independent of age, which did not vary between groups, *F*(3,59) = 0.01, *p* = 0.99. The sample size (*N* = 63) was deemed appropriate because effects sizes tend to be strong and results replicable in research on chess players’ expertise (e.g., Gobet et al., 2004). Ethics approval was granted by Brunel University, UK.

### Task and material

Each position was shown for 5 s, from the viewpoint of white. Next, players were required to evaluate it using the ordinal scale presented in Table 1. This scale is well established in the chess world and is for example used in chess books, search engines, and databases. It is, therefore, an ecological measure.

**Table 1:** Standard evaluations of chess positions

Chess symbol	Meaning
+–	White has a decisive advantage
±	White has a clear advantage
$\underline{\underline{ + }}$	White has a small advantage
=	The position is equal
$\overline{\overline{ + }}$	Black has a small advantage
$\mp$	Black has a clear advantage
−+	Black has a decisive advantage

In total, 56 positions were selected from experts’ (Elo > 2000) games in a commercially available database (Chessbase, 2011). These positions were a representative sample of the kind of positions that players meet in games. Positions were selected randomly from games played by highly ranked professionals. The Elo ratings did not differ, *t*(110) = − 0.891, *p* = 0.375, between the players with white (*M* = 2595.86 Elo; SD = 68.16 Elo) and black pieces (*M* = 2607.11 Elo; SD = 65.44 Elo). Games were selected from recent tournaments to ensure that participants cannot know their evaluation (older games are regularly analyzed and published). Games were deliberately not selected from the strongest players in the world, since those are usually analyzed and immediately published worldwide; thus, participants might have known the published evaluations, biasing our results. The positions were presented as is usual in chess books and software, with white on the bottom half of the board. To avoid attentional biasing, the king was never in check or at the risk of being checkmated. The positions used can be found in the online supplementary material.

The material implemented the seven levels of balance presented in Table 1 and two levels of complexity. The evaluation of the positions was performed using one strong chess program (Fritz 12) and was used as a reference to establish the correctness of the players’ evaluations. As noted above, complexity was operationalized as the number of pieces in a position.

For each of the seven levels of evaluation, we selected four simple and four complex positions. Simple positions had on average 16.04 pieces (SD = 2.46) and complex positions had on average 25.43 pieces (SD = 2.47), *t*(54) = 14.28, *p* < 0.001. In line with the idea that more pieces generate more dynamical complexity, the average number of moves that could be played in simple positions (*M* = 29.36, SD = 8.18) is less than the number of moves that could be played in complex positions (*M* = 40.11, SD = 7.22), *t*(54) = − 5.216, *p* < 0.001. Eight other positions were added to the pool of 56 positions for compatibility with another experiment. The data from these positions were excluded from the analysis of this paper. The positions were saved as images of 456 × 456 pixels.

### Procedure

Participants were contacted by email before international tournaments, informing them of an ongoing research project. They were also approached directly during the tournaments. For professional players, we approached their agent to come to an agreement. All players gave informed consent and were paid for their participation.

After consenting to participate, the players were asked to provide their age, gender and chess rating. The task was completed face to face in one single session. One of the authors was present to answer any question that would be raised by the participants. The instructions for the evaluation task were given verbally and also displayed on the screen. Participants underwent five practice trials to ensure that they were familiar with the chess notation and were at ease with the computer and the response format used. Each trial began with a black mask; the position was then displayed for 5 s. Next, the screen was cleared and the Likert scale with the seven possible evaluations was displayed until the participant provided a judgment. Evaluation and response time were recorded for each trial. The order of positions was randomized for each participant. Players were instructed to provide a judgment as quickly as possible after the display of the Likert scale. There was no time limit for giving an answer, but the players were in general fast (see results below).

### Preprocessing of response times and evaluation errors

For each player, the average response time was calculated. Then, trials with response times (RTs) shorter than 200 ms or longer than the mean plus three standard deviations were discarded (37 out of 3528 trials). The resulting response times were submitted to a log transformation before running the analysis of variance (ANOVA). However, to ease the understanding of the results, response times will be reported in seconds. With respect to position evaluation, the Likert scale was translated into a numerical scale ranging from − 3 (Black has a decisive advantage) to 3 (White has a decisive advantage). For each trial, evaluation error was operationally defined as the absolute distance between the evaluation provided by the computer program and that provided by the player (the smaller the distance, the higher the accuracy).

### Statistical analyses

Response times and accuracy were analyzed separately by using the general linear model. The factorial design with four levels of skill (between-subject), two levels of complexity (within-subject) and seven levels of balance (within-subject) was entered in a full-factorial analysis of variance. The model allowed estimating the influence of the main factors of expertise, complexity, and balance separately, plus estimating the impact of their interactions. We carried out the analysis first on response time and then, separately, on evaluation errors. The statistical analyses were carried out with IBM SPSS^®^.

## Results

### Analysis of response times

Table 2 reports the average response times for each of the experimental conditions. Fifty-eight percent of the RTs were shorter than 2.5 s, only 6.01% of the RTs were longer than 5 s, and only 0.06% were longer than 10 s. These results confirm that the players followed the instructions closely.

**Table 2:** Mean response time (ms) and standard error (ms) for each experimental condition

Complexity	Balance	Skill			
		Candidate	Masters	International	World class
Simple	−+	2517.15 (195.98)	2504.6 (148.34)	2507.67 (231.16)	2218.14 (216.45)
	−/+	2672.97 (244.94)	2828.28 (227.79)	2617.65 (221.33)	2653.68 (312.8)
	=+	2710.81 (206.35)	2540.24 (174.02)	2764.62 (252.90)	2508.41 (174.81)
	=	2532.77 (297.27)	2526.61 (180.02)	2444.84 (219.26)	2445.52 (229.15)
	+=	2631.66 (198.95)	2845.74 (189.22)	2652.66 (169.88)	2899.61 (315.81)
	±	2527.49 (205.71)	2466.84 (168.94)	2701.37 (251.69)	2267.22 (241.89)
	+−	2679.1 (252.61)	2787.68 (202.26)	2672.85 (211.80)	2300.24 (215.98)
Complex	−+	2472.38 (202.61)	2677.86 (164.38)	2820.36 (216.17)	2580.37 (298.21)
	−/+	2769.18 (202.15)	2993.75 (217.12)	2718.23 (216.55)	2886.22 (272.51)
	=+	2856.89 (246.34)	2660.89 (198.56)	2682.24 (189.15)	2654.79 (295.84)
	=	2693.56 (245.93)	2722.24 (161.7)	2362.98 (140.38)	2478.56 (252.97)
	+=	2587.91 (259.60)	2526.01 (115.67)	2442.46 (215.06)	2456.19 (256.27)
	±	2695.44 (245.76)	2383.12 (154.62)	2512.73 (206.99)	1993.15 (150.59)
	+−	2703.62 (232.49)	2502.03 (162.39)	2242.72 (115.02)	2539.44 (231.06)

−+ means decisive black advantage, −/+ clear black advantage, =+ small black advantage, = equal position, += small white advantage, ± clear white advantage, and +− decisive white advantage

Log-transformed RTs were entered in a mixed ANOVA with balance (decisive white advantage, clear white advantage, small white advantage, equal position, small black advantage, clear black advantage, and decisive black advantage) and complexity (simple vs. complex) as within-subject factors and skill (Candidate masters, Masters, International players, and World-class players) as between-subject factor.

As reported in Table 3, only balance was significant out of the three main factors. The only interaction that was significant was balance × complexity, with a linear trend*, F*(1,59) = 14.57, *p* < 0.001, MSE < 0.01. Importantly, RTs did not vary across skills levels, and skill did not interact with any of the other factors.

**Table 3:** Results of the ANOVA on response times

Factor	Degrees of freedom		*F*	MSE	*η*^2^
	Numerator	Denominator			
Balance	6	354	5.763**	0.007	0.089
Complexity	1	59	0.501	0.009	0.008
Balance × complexity	6	354	3.425*	0.006	0.055
Skill	3	59	0.713	0.185	0.035
Balance × skill	18	354	1.324	0.007	0.063
Complexity × skill	3	59	0.447	0.009	0.022
balance × complexity × skill	18	354	0.893	0.006	0.043

*F value is significant at *p* < 0.05, **F value is significant at *p* < 0.01. MSE is the mean square error

### Analysis of evaluation errors

**Fig. 1:**
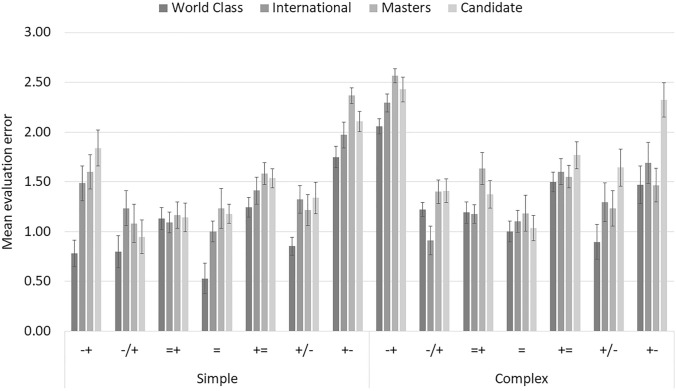
Mean evaluation error per experimental condition. Bars are standard error of the mean. Note: − + means decisive black advantage, −/+ clear black advantage, =  + small black advantage, = equal position, +  = small white advantage, ± clear white advantage, and +—decisive white advantage

As mentioned above, the accuracy measure, is the difference between the players’ estimate of the situation and the computer evaluation of the situation. The smaller the difference, the more accurate players were. Figure 1 shows judgment accuracy for the four skill levels as a function of balance and complexity.

A mixed ANOVA was run on evaluation errors with balance and complexity as within-subject variables and skill level as a between-subject variable. The results are reported in Table 4.

**Table 4:** Results of the ANOVA on evaluation error

Factor	Degrees of freedom		*F*	MSE	*η*^2^
	Numerator	Denominator			
Balance	1	59	43.138**	2.190	0.422
Complexity	1	59	34.271**	0.245	0.367
Balance × complexity	1	59	14.283**	1.737	0.195
Skill	3	59	14.558**	0.489	0.425
Balance × skill	18	354	1.247	2.190	0.060
Complexity × skill	3	59	3.256*	0.245	0.142
Balance × complexity × skill	3	59	2.100	1.737	0.096

*F value is significant at *p* < 0.05, **F value is significant at *p* < 0.01. MSE is the mean square error

Lower bound correction was applied whenever the sphericity assumption was not met (as per Mauchly’s test). All three main factors proved to significantly influence the accuracy of chess players’ judgments. Expectedly, skill had a huge effect on evaluation error: candidate masters (*M* = 1.58; SD = 0.72), masters (*M* = 1.52; SD = 0.74), international players (*M* = 1.39; SD = 0.68), and World-class players (*M* = 1.17; SD = 0.64). With respect to complexity, more accurate judgements were provided for simple positions (*M* = 1.31; SD = 0.68) compared to complex positions (*M* = 1.50; SD = 0.72). Balance also affected evaluation error, with a marked quadratic trend showing that accuracy diminishes when evaluations become more extreme. Figure 1 pictures how evaluation error varies across conditions, revealing the quadratic trend both for simple and complex positions.

The two-factor interactions were: complexity × skill, *F*(3,59) = 3.25, *p* = 0.03, MSE = 0.24, *η*^2^ = 0.14; balance × skill, *F*(3,59) = 1.25, *p* = 0.30, MSE = 2.19, lower bound corrected; and complexity × balance, *F*(6,354) = 14.28, *p* < 0.01, MSE = 0.29*,* *η*^2^ = 0.19 with a linear trend, *F*(1,59) = 65.90, *p* < 0.001. MSE = 0.27.

The triple interaction complexity × balance × skill was not significant, *F*(3,59) = 2.10, *p* = 0.11, MSE = 1.74*,* *η*^2^ = 0.10.

### Predicting evaluation error with Elo rating

**Fig. 2:**
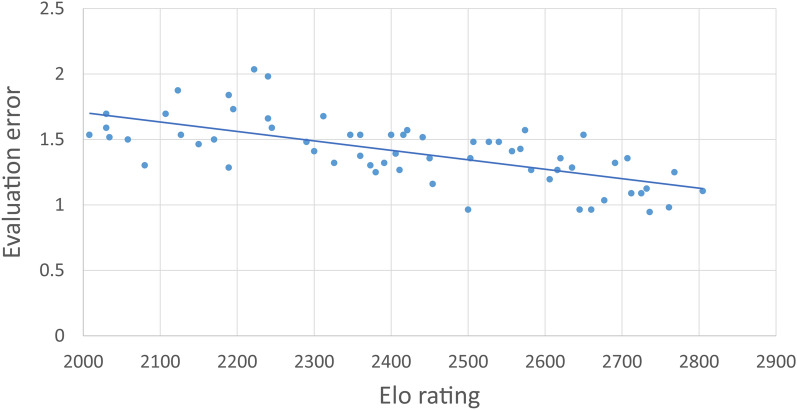
Evaluation error as a function of Elo rating

In a further effort to estimate the effect on skill on accuracy, Elo rating was used as a regressor to predict evaluation error (see Fig. 2). The analysis revealed that Elo rating accounted for 44% of the variance in evaluation error (*r* = 0.664, *p* < 0.001).

1 $${\text{Evaluation error}} = - 0.000{721} \times {\text{Elo}} + {3}.{148}{\text{.}}$$

The coefficient of Eq. 1 indicates that the relationship between Elo rating and evaluation error is negative, supporting the idea that more knowledge provides better intuition. Extrapolating from the data, Eq. 1 indicates that a zero-evaluation error, and thus perfect intuition, is reached with an Elo of 4366. Currently, the best player of the world, world champion Magnus Carlsen, has an Elo of 2859 (International Chess Federation, https://ratings.fide.com/top.phtml?list=men, November 2022).

It could be the case that Eq. 1 is unduly influenced by choices made after long RTs. To test this possibility, we partitioned the evaluation error data in five bins (upper bounds are open): 0–1 s, 1–2 s, 2–3 s, 3–4 s and 4–5 s, and then regressed evaluation error on skill (see Table 5). The results show that skill was a statistically predictor of evaluation error with RTs between 0 and 1 s, 1 and 2 s, and 2 and 3 s, and 3 and 4 s, but not between 4 and 5 s. Thus, we conclude that long RTs did not overly affect Eq. 1.

**Table 5:** Linear regression analyses of evaluation error as a function of skill, with data partitioned using RTs

Model summary						Parameter estimates	
Bin	*R* square	*F*	*df*1	*df*2	Sign	Constant	Coefficient
0–1 s	0.151	4.631	1	26	0.041	5.760	− 0.002
1–2 s	0.189	14.210	1	61	0.001	2.901	− 0.001
2–3 s	0.171	12.358	1	60	0.001	2.968	− 0.001
3–4 s	0.153	10.148	1	56	0.002	3.945	− 0.001
4–5 s	0.005	0.242	1	46	0.625	2.100	0.000

All figures rounded at three decimal places

## Discussion

The aim of this article was to test Dreyfus and Dreyfus’s (1986) and Gobet and Chassy’s (2008, 2009) predictions that experts rapidly reach a holistic understanding of a situation, as measured by chess players’ ability to evaluate a position rapidly. Additional aims were to establish whether complexity and balance affect the quality of evaluations and to quantify the relationship between skill and evaluation.

Altogether, the results support Dreyfus and Dreyfus’s (1986) and Gobet and Chassy’s (2008, 2009) contention that experts understand situations holistically very rapidly. The results show that, in a sample of chess experts ranging from candidate masters to world-class grandmasters, a skill effect in evaluation error emerged after looking at a chess position for 5 s only. A regression analysis quantified the relationship between skill and evaluation error by showing that skill accounted for nearly 44% of the variance in evaluation error. Our results are thus in stark contrast with Moxley et al.’s (2012), who found that skill accounted for only 15% of the move quality. Beyond our contention that evaluation is a better measure of intuition than move choice, other factors might have been operating: our task (5 s) was shorter than Moxley et al.’s (5 min), we used a more standardized method (in particular, computer presentation), and our instrument had higher reliability given that it had a larger number of items. Another key difference with previous studies is the large span of expertise we probed and the very high level of our sample, the weakest of our participants being stronger that the best players in Holding (1979) and Charness (1981). The combination of refinements in the methods and the technologies now available have allowed probing intuition with unprecedented precision. It is important to note that there was no speed-accuracy trade-off, as shown by the lack of skill effects with response times, so the better intuition of experts could not be attributed to an increased processing time. As predicted by our memory-based theory (Chassy & Gobet, 2011), intuition is largely dependent upon domain-specific knowledge, the quantity of which determines the skill of a player and the accuracy of their intuition.

The main effect of complexity, where the accuracy of evaluation was worse with complex than with simple positions, shows that the holistic representation of the position is built incrementally, as argued by Gobet and Chassy (2008, 2009), and not immediately, as claimed by Dreyfus and Dreyfus (1986). When complexity increases, the amount of knowledge necessary to build an accurate representation of the situation increases exponentially; the likelihood of building a correct depiction of the details decreases and so does the quality of intuition. The other main effect, balance, was significant too. The quadratic trend shows that intuition is less accurate when situations become extreme (i.e., clear win for white or black). We cannot rule out the possibility that the trend results from the relatively small sample of positions per condition. Yet, the relative inaccuracy of intuition in extreme situations deserves to be investigated to clarify the cognitive mechanisms that blur intuition. To address the sampling limit, we suggest that future studies use a smaller range of balance with a large sample of positions for extreme situations. The quadratic trend of balance is in line with Gobet and Chassy’s (2008, 2009) theory. It is explained by the fact that extreme situations occur unfrequently and thus do not constitute core knowledge for experts. For example, there is no point in developing a deep understanding of a series of losing situations; it is sufficient to know that it is lost as shown by the fact that most players resign without being checkmated.

The fact that intuition discriminates between players of different skill levels asked to make decisions very rapidly does not mean that look-ahead search is unimportant in chess expertise. A substantial body of evidence, starting from De Groot (1978), has shown that players, when they are not under extreme time pressure and when they face non-routine problems, engage in considerable search. In particular, skill differences have been documented in the statistics of search (e.g., depth of search and number of reinvestigations; Gobet, 2016; Gobet et al., 2004; Holding, 1985). Based on an analysis of the distributions of move times and blunders, Chang and Lane (2016) found that stronger players search more than weaker players even in speed chess (5 min per player for the entire game), sometimes spending more than 1.5 min on one move. Overall, the data do not support the hypothesis that a rapid, unconscious and intuitive system operates initially, followed if necessary by a slow, conscious and analytical system, as proposed by Kahneman (2011). Rather, they suggest that pattern recognition and search are nearly always interleaved when solving problems in chess and in other domains, with intuition playing a key role in all stages of problem solving (Campitelli et al., 2014; De Groot, 1978; Gobet, 1997, 2002; Gobet & Simon, 1998). Our results, which highlight the rapidity and holistic nature of intuition, support this mechanism.

### Possible objections

A possible objection is that 5 s is more than enough time to assess a position using *explicit* criteria. Chess manuals provide advice about how to evaluate a position, typically by comparing white and black along the following criteria: (a) king safety; (b) material; (c) piece activity and mobility; (d) pawn structure (a relatively long-term factor); (e) space advantage; (f) control of the center; and (g) initiative/attack. It is fairly obvious that at least some of these factors take more than 5 s; for example, comparing the material between the two sides involves counting, in a typical middle-game position, up to 24 pieces. Given that, for British English, the typical speaking rate is four syllables per second (Cruttenden, 2014), simply counting the pieces would clearly take more than 5 s. In addition, manuals are typically silent about how these criteria should be combined. Does a space advantage compensate for a weak king position? The fact is that highly skilled chess players very rarely use such a declarative and explicit way of evaluating a position (see, e.g., De Groot, 1978). Similarly, using broad themes such as a good knight versus a bad bishop in most cases is not sufficient to evaluate a position, both because such themes can be subdivided in many sub-themes and because they will have to be combined with other criteria, as just mentioned.

## Conclusion

This study has quantitatively shown that an intuitive understanding of the whole problem situation distinguishes weaker and stronger experts in the very first seconds. As noted in the introduction, it has been elusive to empirically support, let alone quantify, expert intuition in most domains of expertise. An important task for further research will be to replicate our results quantitatively in other domains where experts’ intuition is crucial, and not to rely only on qualitative reports, as it has been predominantly the case.

## Footnotes

1 Chess players’ skill is rated along a continuous scale called the Elo rating (Elo, 1978). After each competitive game, a player’s rating is adjusted as a function of performance (win, loss, or draw) and the rating difference between the two opponents. Experts have an Elo rating of 2000 or above. Below 2000 Elo, the following classes are used: Class A (1800–2000), Class B (1600–1800) and so on.
